# Supplementary material for: SARS-CoV-2-specific humoral and cellular immunity assessment in Peruvian vaccinated population: a cross-sectional study
Source: PeerJ. 2025 Jul 15;13:e19651. doi: 10.7717/peerj.19651 (PMC12273700; doi:10.7717/peerj.19651)
Supplement: Supplemental Information 3 [file peerj-13-19651-s003.doc]

**STROBE Statement Checklist for Cross-Sectional Studies**

**Manuscript Title:** SARS-CoV-2-specific humoral and cellular immunity assessment in Peruvian vaccinated population: A cross-sectional study

This checklist follows the STROBE guidelines for reporting observational studies. Below, we indicate the location in the manuscript where each item is addressed. This checklist has been completed to ensure compliance with STROBE reporting standards for cross-sectional studies.

|  | Item No | Recommendation | Checklist |
| --- | --- | --- | --- |
| **Title and abstract** | 1 | (*a*) Indicate the study’s design with a commonly used term in the title or the abstract | Line 1-3 |
|  |  | (*b*) Provide in the abstract an informative and balanced summary of what was done and what was found | Line 23 -43 |
| Introduction | | |  |
| Background/rationale | 2 | Explain the scientific background and rationale for the investigation being reported | Line 45-93 |
| Objectives | 3 | State specific objectives, including any prespecified hypotheses | Line 93-96 |
| Methods | | |  |
| Study design | 4 | Present key elements of study design early in the paper | Line 99-104 |
| Setting | 5 | Describe the setting, locations, and relevant dates, including periods of recruitment, exposure, follow-up, and data collection | Line 99-104 |
| Participants | 6 | (*a*) Give the eligibility criteria, and the sources and methods of selection of participants | Line 102-104 |
| Variables | 7 | Clearly define all outcomes, exposures, predictors, potential confounders, and effect modifiers. Give diagnostic criteria, if applicable | Line 118-140 |
| Data sources/ measurement | 8* | For each variable of interest, give sources of data and details of methods of assessment (measurement). Describe comparability of assessment methods if there is more than one group | Line 127-174 |
| Bias | 9 | Describe any efforts to address potential sources of bias | Line 150-155 |
| Study size | 10 | Explain how the study size was arrived at | Line |
| Quantitative variables | 11 | Explain how quantitative variables were handled in the analyses. If applicable, describe which groupings were chosen and why | Line 176-182 |
| Statistical methods | 12 | (*a*) Describe all statistical methods, including those used to control for confounding | Line 177-185 |
|  |  | (*b*) Describe any methods used to examine subgroups and interactions | Line 177-185 |
|  |  | (*c*) Explain how missing data were addressed | Line 177-185 |
|  |  | (*d*) If applicable, describe analytical methods taking account of sampling strategy | NA |
|  |  | (*e*) Describe any sensitivity analyses | NA |
| Results | | |  |
| Participants | 13* | (a) Report numbers of individuals at each stage of study—eg numbers potentially eligible, examined for eligibility, confirmed eligible, included in the study, completing follow-up, and analysed | Line 188-190 |
|  |  | (b) Give reasons for non-participation at each stage | NA |
|  |  | (c) Consider use of a flow diagram | NA |
| Descriptive data | 14* | (a) Give characteristics of study participants (eg demographic, clinical, social) and information on exposures and potential confounders | Line 188-198 |
|  |  | (b) Indicate number of participants with missing data for each variable of interest | Line 197-198 |
| Outcome data | 15* | Report numbers of outcome events or summary measures | Line 190-198 |
| Main results | 16 | (*a*) Give unadjusted estimates and, if applicable, confounder-adjusted estimates and their precision (eg, 95% confidence interval). Make clear which confounders were adjusted for and why they were included | Line 200-244 |
|  |  | (*b*) Report category boundaries when continuous variables were categorized | Line 200-244 |
|  |  | (*c*) If relevant, consider translating estimates of relative risk into absolute risk for a meaningful time period | Line 200-244 |
| Other analyses | 17 | Report other analyses done—eg analyses of subgroups and interactions, and sensitivity analyses | Line 237-244 |
| Discussion | | |  |
| Key results | 18 | Summarise key results with reference to study objectives | Line 373-380 |
| Limitations | 19 | Discuss limitations of the study, taking into account sources of potential bias or imprecision. Discuss both direction and magnitude of any potential bias | Line 367-369 |
| Interpretation | 20 | Give a cautious overall interpretation of results considering objectives, limitations, multiplicity of analyses, results from similar studies, and other relevant evidence | Line 262-365 |
| Generalisability | 21 | Discuss the generalisability (external validity) of the study results | Line 380-384 |
| Other information | | |  |
| Funding | 22 | Give the source of funding and the role of the funders for the present study and, if applicable, for the original study on which the present article is based | Line 392-394 |

*Give information separately for exposed and unexposed groups.
